# Unveiling the Uncommon: A Case Report of Horner's Syndrome as a Rare Glimpse Into Giant Cell Arteritis

**DOI:** 10.1155/crnm/2503963

**Published:** 2024-12-10

**Authors:** Emily Barr, Justine Levesque, John Badir, Randall Dunston, Tamra Ranasinghe

**Affiliations:** ^1^Wake Forest School of Medicine, Winston-Salem, North Carolina, USA; ^2^Department of Neurology, Wake Forest School of Medicine, Winston-Salem, North Carolina, USA; ^3^Department of Neurology, Mayo Clinic, Phoenix, Arizona, USA

**Keywords:** GCA, Horner's syndrome, MR angiography, vessel wall imaging

## Abstract

Giant cell arteritis (GCA) is an inflammatory vasculitis affecting large and medium-sized arteries, leading to complications such as arterial dissection, blindness, and stroke. Rarely, GCA presents with Horner's syndrome due to sympathetic neuron involvement from arterial inflammation. This case report discusses an 82-year-old female with hypertension, atrial fibrillation, and arthritis who presented with a 24 h history of right eye ptosis, blurred vision, dizziness, and aching eye pain. She had a mild headache and tenderness over the right temporomandibular joint but no temporal artery tenderness. Examination revealed right eye ptosis and miosis, indicative of Horner's syndrome, with no other neurological deficits. Lab results showed elevated ESR (68 mm/h) and CRP (16 mg/L). MRI with contrast revealed mild to moderate stenosis and enhancement in bilateral MCAs and basilar artery with inflammation in the right distal extracranial ICA, suggesting an inflammatory process. The patient was started on prednisone 40 mg daily. A temporal artery biopsy confirmed GCA with characteristic histopathological findings. Her prednisone dosage was increased to 60 mg/day, and she was started on tocilizumab. This case underscores the need to consider GCA in patients with Horner's syndrome and the importance of vessel wall imaging, as early corticosteroid treatment can prevent complications like vision loss and stroke.

## 1. Introduction

Giant cell arteritis (GCA) is an inflammatory vasculitis with an estimated incidence of 1 in 18–29 cases per 100,000 people over the age of 50 in the United States [1, 2]. GCA typically occurs in individuals over the age of 50 with a median age at diagnosis of 73 years old and a frequency twice as high in women as in men [3]. It is a complex rheumatological disorder, characterized by granulomatous inflammation affecting large- and medium-sized arteries, and is often referred to as temporal arteritis due to its frequent involvement of the branches of the external carotid artery. GCA can present with a variety of symptoms including headache, jaw claudication, temporal scalp tenderness, visual deficits, and constitutional symptoms. The most common severe complication is blindness from optic nerve ischemia. The etiology of GCA is multifactorial, encompassing both genetic predisposition and immune dysregulation.

The diagnostic criteria for GCA as described by the American College of Rheumatology are based on both clinical and imaging criteria as outlined in Table 1 [1].

Due to the risk of severe complications, GCA is a medical emergency and requires prompt diagnosis and treatment. However, prompt diagnosis can often be challenging due to overlap of symptoms with other common conditions [4]. We present a patient who exhibited right-sided incomplete Horner's syndrome for 24 h and was subsequently found to have GCA. Rarely, GCA patients present with Horner's syndrome due to inflammation of internal carotid arteries which are in close proximity to the third order neurons of the sympathetic chain. Additionally, this case highlights extracranial and intracranial vessel involvement in GCA.

## 2. Case Presentation

An 82-year-old female with a history of hypertension and atrial fibrillation on dabigatran and aspirin presented with right eye ptosis for 24 h. On arrival, her blood pressure was 162/91 mmHg, heart rate was 65 beats per minute, and respiratory rate was 20 breaths per minute, and she was afebrile. She reported a constant drooping of her right eyelid associated with blurred vision, right eye pain, and dizziness. On exam, right temporomandibular tenderness was elicited without temporal scalp tenderness, palpable pulsation, or hardening of the superficial temporal artery. Her neurological exam demonstrated mild right eye ptosis and miosis without anhidrosis suggestive of incomplete Horner's syndrome. No other focal neurologic deficits were elicited. Initial serum blood counts and basic metabolic panel were unremarkable, but notably ESR was elevated at 68 mm/h and CRP elevated at 16 mg/L.

**Figure 1:**
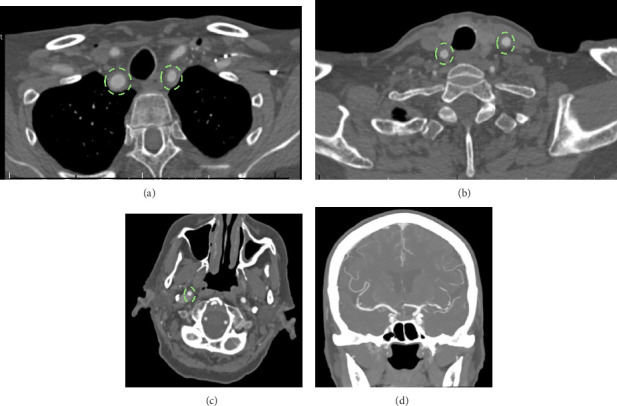
(a, b) CTA head and neck demonstrating diffuse mural thickening of bilateral subclavian and carotid arteries (dotted outline). (c) CTA neck demonstrating mural thickening of the right distal internal carotid artery. (d) CTA head demonstrating bilateral MCA stenosis.

**Figure 2:**
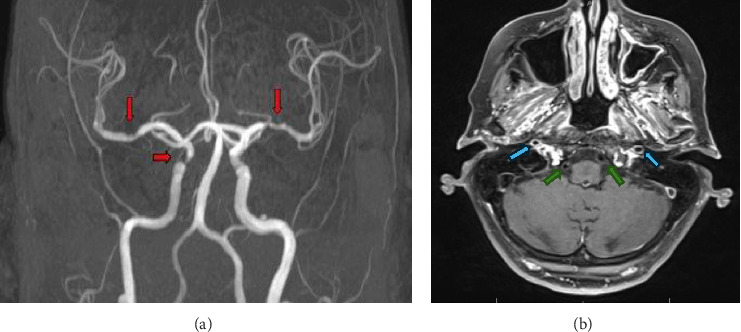
(a) MRA brain demonstrating bilateral M1 segment (downward arrow) and paraclinoid right internal carotid artery (horizontal arrow) narrowing. (b) MRA brain imaging demonstrating concentric enhancement of bilateral internal carotid arteries (blue arrows) and nonenhancing vertebral arteries (green arrows).

**Figure 3:**
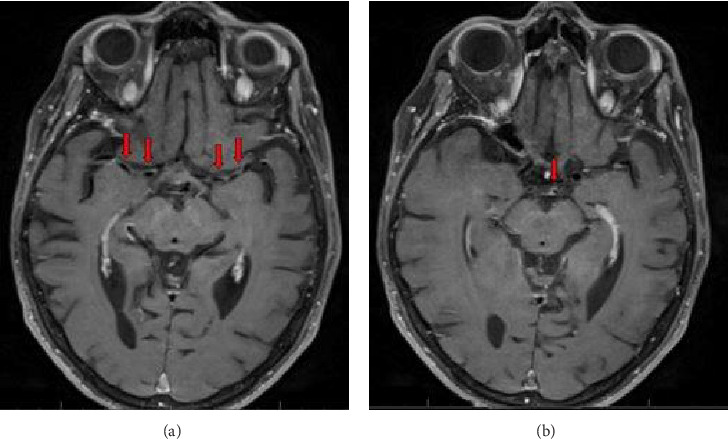
(a) MRI brain T1 post-contrast image demonstrating enhancement of bilateral MCA vessel walls. (b) MRI brain T1 post-contrast image demonstrating enhancement of the basilar artery vessel wall.

CT angiography (CTA) neck demonstrated diffuse mural irregular thickening of the proximal great vessels including the subclavian arteries, extending up to carotid artery bifurcation, as well as the distal right internal carotid artery (Figure 1). CTA head demonstrated multifocal luminal narrowing in the left greater than right middle cerebral artery MCA (Figure 1). MRI brain with contrast and MR angiography (MRA) with contrast vessel wall imaging demonstrated moderate stenosis over bilateral MCAs in the M1 segments (Figure 2) with vessel wall enhancement in the bilateral M1s, the basilar artery, and petrosal extracranial internal carotid artery (Figure 3), suggestive of an inflammatory process. No acute ischemic stroke or hemorrhage was identified.

Given the concern for GCA, the patient was initiated on oral prednisone 60 mg daily. Outpatient temporal artery biopsy was performed and demonstrated focal disruption of the internal elastic lamina, multinucleated giant cells within the media, lymphocytic inflammatory infiltrates in perivascular soft tissue, and focal medial calcifications, confirming the diagnosis of GCA. At a two-week follow-up appointment, the patient endorsed improvement of blurred vision, ptosis, and jaw pain with normalization of inflammatory markers. Later the patient was initiated on weekly subcutaneous tocilizumab 162 mg. At one-month follow-up visit, her Horner's syndrome had resolved [1].

## 3. Discussion

Horner's syndrome is a rare presentation of GCA and has been discussed in the medical literature. It is suggested that the pathophysiology behind an incomplete Horner's syndrome presentation in GCA is due to inflammatory lesions occurring in the internal carotid artery that led to decreased perfusion or damage to the sympathetic fibers. This damage can occur either through direct involvement of the granulomatous tissue with the sympathetic nerve fibers surrounding the artery or more likely through ischemic damage caused by the blockage of the blood vessels (vasa nervorum) supplying the nerve fibers [5].

To our knowledge, this is one of a select few case reports of a GCA patient presenting with Horner's syndrome with evidence of abnormal vessel wall imaging. This case also adds to the limited literature that suggests GCA affects intercranial arteries and can present with Horner's syndrome as a primary symptom. Prior to our case report, published literature showing Horner's syndrome as a presenting symptom of GCA is limited to that of a handful of case series and case reports with outcomes described in Table 2.

Aside from the above case reports, the most recent literature suggesting involvement of Horner's syndrome in GCA with cranial imaging is limited. Prior published case reports describe patients presenting with postganglionic Horner's syndrome and GCA, yet none reported findings of vessel wall enhancement on neuroimaging. Some case reports describing GCA and Horner's syndrome were published prior to advanced neuroimaging techniques [7, 8] limiting their application to cases today.

Modern advancements in neuroimaging offer a tremendous advantage in the prompt diagnosis of vasculitis. Many different imaging modalities are available with differing sensitivities and specificities as outlined in Table 3. Sverdlichenko et al. conclude their report by suggesting neuroradiological evaluation of the entire sympathetic chain is key in patients > 50 years old who present with new-onset Horner's syndrome, along with review of the pertinent GCA history, symptomatology, and trending of inflammatory markers [6]. Our report supports this assertion given our patient's MRI brain demonstrated mild to moderate stenosis and enhancement over the bilateral MCAs and basilar artery, suggestive of an inflammatory process. This finding demonstrates the utility of MRI and MRA brain and neck to assess arterial supply for patients exhibiting symptoms of GCA and Horner's syndrome. There is evidence to support the use of gadolinium-enhanced MR improved motion sensitized driven equilibrium (iMSDE) in patients with GCA as this type of neuroimaging can enable more direct visualization of the vessel wall and possible mural enhancement because of reduced blood flow artifact [16].

The most effective therapy to date has been the use of high-dose glucocorticoids such as prednisone at a dose of 40–60 mg/day administered in a single daily dose. This regimen has been proven to reduce the chance of visual loss when given early in diagnosis [17]. Care should be taken when performing advanced vessel wall imaging such as MRI or ultrasound after starting steroid treatment as studies suggest decreased sensitivity in detecting GCA; therefore, imaging should be performed as soon as possible within the first few days of treatment [18]. There is also evidence to suggest that antiplatelet therapy, such as low-dose aspirin, may prevent ischemic events, such as stroke and vision loss, in patients with GCA [19]. In patients who are at increased risk for adverse effects related to glucocorticoids, guideline therapy recommends adding tocilizumab, an IL-6 inhibitor that works by decreasing the formation of acute phase reactants which trigger inflammation in diseases like GCA. Current dose recommendations of tocilizumab include 162 mg subcutaneous injection weekly or as an intravenous infusion combined with glucocorticoids monthly administered at 60–80 mg/kg [20].

## 4. Conclusion

In patients over the age of 50 years old who have symptoms of GCA and present with Horner's syndrome, we recommend checking inflammatory markers and obtaining vessel wall imaging of head and neck vasculature. It is critical that GCA be identified early within the disease process to start necessary treatments to prevent long-term deficits, most notably blindness.

## Figures and Tables

**Table 1:** 2022 American College of Rheumatology/EULAR classification criteria for giant cell arteritis [1].

Age	Clinical presentation	Laboratory findings	Imaging/biopsy
≥ 50 years old (absolute requirement)	- Morning stiffness in shoulders/neck (+2) - Sudden visual loss (+3) - Jaw or tongue claudication (+2) - New-onset temporal headache (+2) - Scalp tenderness (+2) - An abnormal temporal artery examination (+2)	- Maximum ESR greater than 50 mm/h OR - Maximum CRP greater than 10 mg/liter^2^ (+3)	- Positive temporal artery biopsy or halo sign on ultrasound (+5) - Bilateral axillary involvement on angiography (+2) - Abnormal FDG-PET uptake in the arterial wall throughout the aorta (+2)

*Note:* Considerations for applying these criteria—should be applied to classify the patient as having GCA when a diagnosis of medium- or large-vessel vasculitis has been made, and alternate diagnoses mimicking vasculitis should be excluded to applying the criteria. If the sum of the following 10 categories is ≥ 6, then the patient meets classification for giant cell arteritis.

**Table 2:** Prior case reports of patients with GCA presenting with Horner's syndrome.

Authors	Case	Imaging findings	Conclusions
Sverdlichenko et al. [6]	Two patients	- MRI brain and MRA of the head and neck as well as CT head and CT angiography of the head and neck were all unrevealing - No MRA findings	Postulated that GCA affected the small perforator branches of the vertebral arteries that supply the dorsocaudal midbrain, subsequently resulting in ischemia of the sympathetic chain as it passes through the midbrain
Arunagiri, Santhi, and Harrington [7]	77-year-old	A normal MRI brain	Argued against a preganglionic lesion and therefore the authors were more suspicious of a cavernous sinus lesion

**Table 3:** Vessel wall imaging modalities and their associated findings.

Modalities for vessel wall imaging/diagnosis	Imaging findings associated with GCA [1, 5]	Reported sensitivity (%)	Reported specificity (%)
Ultrasound	Wall swelling (“halo sign”) in cross section temporal artery is diagnostic for acute temporal arteritis	88 [9, 10]	96 [9, 10]
Positron emission tomography/computed tomography (PET/CT)	Hypermetabolism along the vessel wall suggesting large vessel vasculitis	83 [11]	89.6 [11]
Computed tomography angiography (CTA)	Mural thickening with double ring enhancement after an intravenous injection of iodine-based contrast is observed in patients with large vessel vasculitis GCA	73 [12]	78 [12]
MRI	Increased vessel wall thickness and edema with increased mural enhancement on high-resolution post-contrast images	73 [13]	88 [13]
Biopsy	Histopathology shows granulomatous inflammatory process seen along the internal elastic lamina of involved vessels	77 [14]	100 [15]

## Data Availability

The case and imaging data used to support the findings of this study are available from the corresponding author upon request.
